# Publicity and Common Commitment to Believe

**DOI:** 10.1007/s10670-021-00393-x

**Published:** 2021-05-24

**Authors:** J. R. G. Williams

**Affiliations:** University of Leeds, Leeds, UK

## Abstract

Information can be public among a group. Whether or not information is public matters, for example, for accounts of interdependent rational choice, of communication, and of joint intention. A standard analysis of public information identifies it with (some variant of) common belief. The latter notion is stipulatively defined as an infinite conjunction: for *p* to be commonly believed is for it to believed by all members of a group, for all members to believe that all members believe it, and so forth. This analysis is often presupposed without much argument in philosophy. Theoretical entrenchment or intuitions about cases might give some traction on the question, but give little insight about *why* the identification holds, if it does. The strategy of this paper is to characterize a practical-normative role for information being public, and show that the only things that play that role are (variants of) common belief as stipulatively characterized. In more detail: a functional role for “taking a proposition for granted” in non-isolated decision making is characterized. I then present some minimal conditions under which such an attitude is correctly held. The key assumption links this attitude to beliefs about what is public. From minimal a priori principles, we can argue that a proposition being public among a group entails common commitment to believe among that group. Later sections explore partial converses to this result, the factivity of publicity and publicity from the perspective of outsiders to the group, and objections to the aprioricity of the result deriving from a posteriori existential presuppositions.

**Supplementary Information:**

The online version contains supplementary material available at 10.1007/s10670-021-00393-x.

## Introduction

When we are acting alone, there are things we take for granted. I searched for my keys this morning. While I took seriously the possibility of my keys being in the bathroom or the hallway, I took for granted that they were lost somewhere in the house rather than stolen, and took for granted that they were visible, solid and non-explosive.

What we take for granted is held fixed in deliberation. The consequences of a possible act will depend on the background state of the world. One option that was open to me this morning was to search the hall for the keys—that likely had good consequences on the supposition they were in the hall, worse consequences if they were in the bathroom. And notice: it would have wasted precious time if the keys had been stolen, would have been futile if they were invisible, and hazardous if the keys were explosive. In calculating these consequences of a potential act under one supposition or another, I am always prepared to introduce, under that supposition, any proposition I take for granted.

My working hypothesis about what plays this role in deliberation: rational agents take something for granted in practical reasoning if and only if they believe it to be the case.^1^

Acting alone is, however, just a limiting case of action. Often the consequences of my actions depend on what someone else does; the consequences of driving down a street in my black car are quite different if the person waiting in the red car on a side street bides their time, rather than stamping on the accelerator as I pass. My first-personal deliberations, in the general case, need to account for the possible impact of the actions of others. When other people are involved in a decision situation, we can distinguish two ways that each can take a proposition for granted. Both involve being prepared to introduce that proposition under the scope of a range of suppositions. In the first (weak) sense the suppositions in question are just as before: factual suppositions that the agent makes about the background state of the world. In the second (strong) sense the suppositions in question are broader, to include also perspective-switching suppositions “Suppose I were the other driver…” in which the agent emulates the perspective of others, in order to anticipate their likely actions. I take a proposition for granted in the strong sense if I take it to be part of the perspective of everyone in the decision situation. (There are cases where we may treat others as automata, and their actions as naturally arising phenomenon which we predict without considering how they are determined by the other’s psychology. But in the general case, our fix on how others act depends in part on anticipating how things look from their perspective).

Complementary to the deliberative perspective of an agent involved in action, i.e. the insider’s perspective, there is also the perspective of one not involved in the action, i.e. the outsider’s perspective. The outsider looks at a pattern of actions undertaken by each member of a group of which they are not themselves a part, and seeks to understand them. An outsider, observing from an upper window drivers approaching each other on the road below, might say “Out of sight of these two, I can see a flash flood about the engulf the road that they are driving on. Both of these drivers are taking for granted the absence of any danger from flooding–-that’s why they’re continuing to drive onwards rather than fleeing”. The outsider attributes attitudes to the insiders, and can characterize what they take for granted in the weak and the strong sense. But as the case of the flood illustrates, the outsider need not take for granted (even in the weak sense) the propositions they think the insiders take for granted. In the example, the outsider views the drivers as taking a dry road for granted, but she does not take this for granted herself! This paper first examines the attitudes and concepts characteristic of the insider’s perspective, and only after this returns to examine the outsider’s perspective. This order of explanation should be unsurprising; after all, the outsider’s task in explaining why insiders did what they did is to represent the mental states characteristic of the insider’s perspectives on their various actions.

What is it for an insider to “take *p* for granted in the strong sense”? A first observation: that an agent believes something doesn’t make it okay for them to take it for granted in the strong sense. Under the perspective-switching supposition that I am the driver of the red car, I do not add the information that the black car is driven by a nice person who is currently distracted by some philosophical puzzle—all things I myself believe, but which the other has no basis for believing. Nor will I take for granted (in the strong sense) everything that I, in addition to believing myself, believe the other believes. What I believe the other believes is the resource relevant for emulating the other’s factual assumptions—what they take for granted in the *weak* sense. But since the other is also working out what *I’ll* do in this situation, a component of the other’s practical reasoning is what they take for granted in the *strong* sense, and so in emulating their practical reasoning what I may take for granted in the strong sense turns on what they take for granted in that same strong sense.

The upshot of the above is that it isn’t at all obvious how to give an illuminating characterization of what an agent should take for granted in the strong sense that doesn’t itself use the terms “taking for granted in the strong sense”. In the literature on non-isolated decision making, there is a salient candidate for this role, however. This starts with a stipulative definition: for *p* to be commonly believed among a group G is for everyone in G to believe *p*, for all to believe that all believe *p*, for all to believe that all believe that all believe *p*, and so on forever.^2^ Then: what I take for granted is what I believe, and believe to be commonly believed; what you take for granted is what you believe, and believe to be commonly believed.^3^

This proposal has at least the right kind of shape to play the role just outlined. It entails that I believe *p*, and so take it for granted in the weak sense. And if I have this complex combination of beliefs, and believe the immediate consequences of what I believe, I’ll believe that you have an exactly matching combination of beliefs about me. The latter corresponds to the natural thought that in order to take something for granted in the strong sense, I need to be assuming that you are also taking it for granted in the same strong sense. My aim in this paper is to give an argument for a version of this hypothesis, and defend the hypothesis against objections. In step with others, I will end up focusing not on common belief, but on a close variant that I call common commitment to believe, or for short, common belief*.^4^

The first few sections focus on constructing an argument that propositions that are *public among the group* will be commonly believed*—publicity is here a property of propositions which is implicitly defined in terms of taking for granted. I then identify the additional assumptions required to show that what is commonly believed is public, and assess what this teaches us about the identity of the property. After the main argument, I present further principles that allow us to derive a quasi-converse result. The quasi-converse result has an initially puzzling feature (factivity) and I trace this to our initial decision to focus on the insider’s perspective. I also show how one can develop a parallel account of a non-factive notion of publicity, by rerunning the discussion and adapting the premises to the outsider’s perspective. The closing sections then highlight a difference between common belief(*) as I characterize it and the usual formal articulations, and use this to formulate an objection to the upshot of earlier sections, which I then seek to defuse. An Online appendix formalizes the arguments sketched informally in the main text.

## Five Premises, Two Intermediate Conclusions

We are interested in what it is to take some proposition for granted in the strong sense, when engaged in practical reasoning in a non-isolated decision situation involving a group of agents G. But that is a mouthful. I shorten this to “treating *p* as public among G”.^5^ Our question, therefore, is: what is it for an agent to treat *p* as public among group G? The method for answering it is to list a priori constraints on the notion, and see what follows.

First, given the discussion of the previous section, taking a proposition for granted in the strong sense, requires at least that we take it for granted in the weak sense. Our hypothesis was that to take something for granted in the weak sense is to believe it, and so we have the following:

(BELIEF ENTAILMENT)

For all *x,* (if *x* treats *p* as public among G, then *x* believes that *p*).^6^

The quantifier “for all x” is intended to be read unrestrictedly (I will say the same about quantifiers that appear in the other principles introduced below). I intend my premises to capture general a priori truths about *treating as public*, irrespective of who instantiates the attitude.

The reader might pause and wonder whether a restriction of the quantifiers in principles like (BELIEF ENTAILMENT) to members of G is in order. After all, in the previous section, I emphasized that outsiders, reconstructing the practical reasoning of those involved in a non-isolated decision situation, need not believe-true the things that (in their view) the insiders are taking for granted. But this doesn’t imply that BELIEF ENTAILMENT requires restriction; rather, it reflects the fact that outsiders don’t need to *treat propositions as public among G* in order to reconstruct insiders’ reasoning. Outsiders engaged in the reconstructive task might adopt a higher order attitude, for example, believing that every member of G treats-p-as-public-among-G (Sect. 6 examines such questions in detail). There’s no motivation from the previous section for restricting BELIEF ENTAILMENT to insiders.

Even if unmotivated, a version of BELIEF ENTAILMENT with quantifiers restricted to members of G will be strictly weaker than the premises on the unrestricted interpretation I give them. The same will be true of the other premises below. Thus, someone could coherently doubt my premises on their intended unrestricted reading, but accept them on a restricted reading. Further, since my arguments will be formally valid, from a restricted-domain version of the premises, a restricted-domain version of my conclusion would follow. This is a reading available to any reader who continues to prefer to interpret my premises restrictedly, but I will say no more about it.

I propose to study treating-as-public by looking at its correctness conditions, in that objective sense of “correctness” which makes the following a platitude:

(BELIEF CORRECTNESS)

For all *x*, *x*’s belief that *p* is correct iff *p*.^7^

From the previous section, we can extract a necessary condition on something being correctly treated as public. Consider when our group consists of A and B, and A treats *p* as public, but B does not. Then A is disposed, when emulating B’s perspective, to adduce *p* under arbitrary suppositions. But B, in reasoning, will not adduce *p* under arbitrary suppositions. So A is not emulating B’s perspective correctly. However reasonable she was, in these circumstances, she has made a mistake. Generalizing, someone treating something as public is correct only when that same attitude is replicated throughout the group:

(TREATS CORRECTNESS)

For all *x*, *x* treating *p* as public among G is correct only if all members of G treat *p* as public among G.^8^

The three schematic assumptions so far should be generally acceptable. I will assume that they are true. I further assert that they are a priori, though more will be said on the latter point in later sections.

I need two more principles. Each asserts, a priori, a connection between *treating p as public (among G)* and a certain belief, a belief with the content that *it is public that p.* The first of these principles asserts an equivalence in correctness conditions. The other asserts an equivalence in the conditions under which the attitudes are tokened.

(CORRECTNESS EQUIVALENCE)

For all *x, x* is correct in treating *p* as public among G iff *x* is correct in believing that it is public in G that *p*.^9^

(INSTANTIATION EQUIVALENCE)

For all *x*, *x* treats *p* as public among G iff *x* believes that it is public in G that *p*.^10^

The obvious way to motivate both is by motivating an identity: treating *p* as public (among G) *just is* to believe that it’s public (among G) that *p*.

Why accept this identity? We might regard it as a speculative hypothesis about the reduction of one attitude to another. Multiplying irreducible sui generis attitudes endlessly is unattractive, and so it would be theoretically neat if we could identify treating-*p*-as-public-among-G with some belief or other the content of which involves *p* and G. The operator “it is public that” can then be regarded as a placeholder for whatever the missing ingredient is. Such a speculative reductive hypothesis may of course fail, but it is of obvious interest to figure out whether it might be true, and if it is true, what follows.

We might also be led to this identity from a very different starting point. We might think that we already have a good functional fix on what “treating *p* as public” amounts to, one that requires no reduction to belief for legitimation. One then looks to the expressivist tradition which regards various superficially assertoric claims—“murder is wrong”, “if he dropped it, it broke”, “the keys might be in the hall”—as *most fundamentally* expressing attitudes other than belief—planning states, conditional beliefs, or uncertainty, respectively. Contemporary expressivists tend not to deny that such claims *in some derivative sense* express the belief that murder is wrong, etc., but think of belief-talk when it involves the expressivist content as another way of reporting the underlying non-doxastic attitudes. In just this way, one could think of the operator “it is public that” as an expressive device, and an assertoric utterance of “it is public in G that *p*” as expressing resolve to treat-as-public *p* among G, and so be led to the view that a belief that it is public in G that *p* as simply another label for this attitude of treating-as-public *p* among G. This is a view that I personally find attractive.

Just as with the initial three assumptions, I assert that this identity and the two principles that flow from it are a priori true, if true at all. One could, I suppose, think that the identity between treats-as-public and belief is an empirical matter, to be discovered by brain-scans and the like, but I set aside that possibility.

I now put the pieces together (the argument that follows, as well as later ones, are formalized in the technical appendix). BELIEF CORRECTNESS gives us something a priori equivalent to the right hand side of CORRECTNESS EQUIVALENCE. TREATS CORRECTNESS give us an a priori consequence of the other side. Putting this together, the result is the following:

(PUBLIC)

It is public in G that *p* only if everyone in G treats *p* as public among G.^11^

Since CORRECTNESS EQUIVALENCE is a priori, this conditional is a priori.

INSTANTIATION EQUIVALENCE allows us to replace the consequent of PUBLIC with something a priori equivalent, giving us the following a priori conditional:

(INTROSPECTION)

It is public in G that *p* only if everyone in G believes that it is public in G that *p*.^12^

This is the first key result I take away from this section.

Suppose that it is public that *p* among G. Then by PUBLIC everyone in G treats *p* as public among G. And by BELIEF ENTAILMENT everyone in G believes *p*. All this is a priori reasoning from a priori principles, and so we have, a priori:

(PUBLIC BELIEF ENTAILMENT)

It is public that *p* among G only if everyone in G believes *p*.^13^

To sum up: I started with three principles that are intended to be both a priori and uncontroversial. I then added two principles that I assert to be a priori if true, but which are controversial. From this we take forward the following pair of claims which don’t mention “treating as public” at all:

- (1) A PRIORI PUBLIC BELIEF ENTAILMENT
  It is a priori that: it is public in G that *p* only if everyone in G believes *p*.
- (2) A PRIORI INTROSPECTION
  It is a priori that: it is public in G that *p* only if everyone in G believes that it is public in G that *p*.

The original question was: what is it to treat a proposition as public among *G*? In this section, I made an assumption that treating something as public is to have a certain belief involving the concept “it is public in G that”. So we’d have an answer to the original question if we knew the character of this property of propositions *being public among G.* (1) and (2), it turns out, tell us a lot about this property.

## Formal Interlude

It is very common to model belief and common belief in multimodal logics (Fagin et al 1995). Such logics involve a series of belief operators ${B}_{1},\dots ,{B}_{n}$—one for each member of our group. We may define mutual belief $Mp:={M}^{1}p:={B}_{1}p\wedge \dots {B}_{n}p$ (“everyone believes p”) and more generally arbitrary orders mutual belief by ${M}^{k+1}p:=M{M}^{k}p$. Common belief, as introduced earlier, is standardly modelled by the infinite conjunction: $Cp:=\underset{0<i<\infty }{\bigwedge }{M}^{i}p$.^14^

This setting models the group using *n* distinct modal operators, which each map propositions to propositions. One known result in such logics is that if there is an operator P for which $Pp\supset Mp$ and $Pp\supset MPp$ are both valid, then $Pp\supset Cp$ is valid.

The principle (1) from the previous section looks very much like it might be modelled formally by taking $Pp\supset Mp$ to be valid, and similarly (2) looks like it might be modelled formally by taking $Pp\supset MPp$ to be valid. And if so, we have two premises of a formal argument, the informal rendering of whose conclusion is that publicity entails common belief. And no more is needed, apparently, than the results derived a priori from minimal assumptions of the last section.

I will not set out the above result here, or rely on it in any way. I do not presuppose some things which are baked into the standard formal model; as we’ll see in Sect. 7, there are subtle differences between the formulations of the notions (my use of restricted quantification vs. the standard model’s use of long conjunctions turns out to be philosophically substantive). Still, the argument of Sect. 4 should not come as a surprise, given this precedent.

## Publicity Entails Common Commitment to Believe

Stalnaker, Lewis and their followers think that if a person has a belief with the content *p*, and *p* necessarily entails *q*, then the person must have a belief with the content *q* as well.^15^ This is a very surprising claim, and not one I endorse.

There is a closely related claim, however, that is a mere triviality. Say a person is *modally committed to believe p* when *p* is necessarily entailed by something that person believes. Clearly, if a person is modally committed to believe *p*, and *p* necessarily entails *q*, then they are modally committed to believe *q*. This “closure” principle is analytic and a priori, given the way that the target notion was defined. I concentrate on the a priori modality, and so define a person being *committed to believe p* simpliciter iff *p* is a priori entailed by something that person believes. Then we have the following:

- (3) A PRIORI CLOSURE OF COMMITMENT

It is a priori that: if *x* believes *p*, and *q* follows a priori from *p*, then *x* is committed to believe *q*.^16^

We’re going to show that *it being public in G that p* a priori entails:

(i) that everyone in G believes *p*;

(ii) that everyone in G is committed to believe that everyone in G believes *p*;

(iii) that everyone in G is committed to believe that everyone in G is committed to believe that everyone in G believes *p*,

(iv)…

and so forth.

The infinite conjunction (i), (ii), (iii)… I call common belief*, or more wordily, common commitment to believe.^17^ We need to show that a proposition being public in G requires, a priori, that it is commonly believed*. So suppose that it is public in G that *p*. We then argue successively:

- Given the belief entailment principle (1), that everyone believes *p* follows a priori from the assumption that it is public in G that *p*. This establishes clause (i) as an a priori consequence of it being public that *p*, as required.
- From the introspection principle (2), *everyone believes that it is public in G that p* follows a priori from the assumption that is public in G that *p*. In the bullet point immediately above it was demonstrated that *everyone in G believes p* is an a priori consequence of the content of what everyone believes, viz. *it being public in G that p.* So by the closure principle (3), everyone in G is committed to believing that everyone in G believes *p*. This is clause (ii). This derivation just given is a priori, so (ii) is an a priori consequence of it being public that *p*, as required.^18^
- From the introspection principle (2), *everyone believes that it is public in G that p* follows a priori from the assumption that is public in G that *p*. In the bullet point above it was demonstrated that *everyone in G is committed to believing that everyone in G believes p* is an a priori consequence of the content of what everyone believes, viz. *it being public in G that p.* So by the closure principle (3), everyone in G is committed to believing that everyone in G is committed to believing that everyone in G believes *p*. This is clause (iii). This derivation is a priori, so (iii) is an a priori consequence of it being public that *p*, as required.

The pattern of the last two bullet points repeats indefinitely. One can turn this infinite proof into a formal induction in the metalanguage in order to give a finite proof of the result.

Publicity, on my telling, is implicitly defined by the equivalences between (a) beliefs about what propositions are public and (b) the attitude of treating-as-public. From these connections, and independently compelling principles, we extracted two a priori constraints on beliefs about publicity, (1) and (2). By defining “commitment to believe” in the right way, we get (3), and as we have just seen, these together deliver the result that publicity entails common belief*.

To emphasize: I have not shown that publicity entails common belief. Common belief was defined as the infinite conjunction: everyone believes *p*, everyone believes everyone believes *p*, everyone believes everyone believes everyone believes *p*, etc. Common belief follows from common belief* if you add the additional premise that when someone is committed to believe *p*, they in fact do believe *p*. But the result as stated does not require that controversial assumption.^19^

## Factivity and Sufficient Conditions for Publicity

Publicity entails common commitment to believe. That leaves open what more is needed for a proposition to be public. Some might suggest further conditions relating to explicit assertion, or to some manifest jointly-attended-to event, or to reciprocal acknowledgement of publicity.

In this section I argue that when something is a true common belief, then it is public. Granted the latter, nothing that is not entailed by true common belief can be a necessary condition on a proposition being public. Since explicit assertion, manifestly being jointly attended to, etc., are not entailed by a proposition being true and commonly believed, they can’t be required for a proposition to be public.

Why *true* common belief? It turns out that the concept of publicity, when motivated from the insider’s perspective as I have done, is factive. This section explains why this is so. Some have found that result troubling, but they should not—it is a natural result of something we built into the concept from the start. The next section identifies the source of factivity and, by reconsidering the dialectic from the outsider’s perspective, shows how to tweak our starting assumptions to motivate and develop a parallel non-factive notion of publicity (publicity2).

The argument I will give in this section adds two premises to those assumptions used in previous sections. The first identifies a condition that is sufficient for treating something as public. Suppose that *p* is a common belief in G. It follows that an arbitrary group member, Anna, believes *p*. So she takes it for granted in the weak sense. It also follows that she believes that everyone else believes that *p*. So when she thinks of the world from their point of view, she’ll take for granted *p*. She believes that everyone else believes that everyone else believes *p*, and so when she thinks of the world from the point of view of a second group member thinking of the world from the point of view of a third group member, she will take for granted *p*. This iterates indefinitely. I submit that the dispositions just characterized are sufficient for Anna to count as taking *p* for granted in a strong sense, i.e. treating *p* as public. Accordingly, the first premise of the new argument is:

(COMMON BELIEF)

If it is commonly believed in G that *p*, then everyone in G treats *p* as public in G.^20^

The second premise I need is a biconditional strengthening of the earlier TREATS CORRECTNESS. To treat *p* as public among G is to take it for granted in the strong sense, and taking for granted in the strong sense involves both a person-directed component and a world-directed component. The person-directed component means that one holds *p* fixed under suppositions where you take the perspective of others, and it is this that means that it is a condition of correctness that others mirror the attitude, which is captured in TREATS CORRECTNESS. The world-directed component of the attitude means that one holds *p* fixed under suppositions about what the world would be like, were one to do this or that. One would be making a mistake in doing this, if *p* were not in fact the case.^21^ Accordingly, we should strengthen the right hand side of TREATS CORRECTNESS by adding *p* as a conjunct. But nothing more than this is required. The following biconditional is appropriate if these two conditions are jointly sufficient for treating *p* as correct.

(STRONG TREATS CORRECTNESS)

For all *x*, *x* treating *p* as public among G is correct if and only if *p* and all members of G treat *p* as public among G.^22^

Using the premises introduced in Sect. 2, we can argue that *p* is public among G iff x’s belief that *p* is public in G is correct iff it is correct for *x* to treat *p* as public. Adding to this chain of biconditionals, STRONG TREATS CORRECTNESS allows us to derive, a priori:

(STRONG PUBLIC)

It is public in G that *p* if and only if *p* and everyone in G treats *p* as public among G.^23^

STRONG PUBLIC tells us, among other things, that publicity is factive. So not only does publicity entail common belief*, it entails *p* is true and commonly believed*. That was the one of the results I promised at the start of this section. Further, if we put (COMMON BELIEF) and (STRONG PUBLIC) together, we have the central result I promised at the start of the section: *p* is public in G if *p* is *true and commonly believed among G.*

Pulling this all together, we have the result that true common belief entails publicity, which entails true common belief*. Under the assumption that common belief* entails common belief (which, remember, follows from the Stalnaker-Lewis assumption that belief is closed under single-premise a priori entailment), then we would have pinned down publicity completely: something is public iff it is true common belief. If the Stalnaker-Lewis assumption fails, as I think it does, then we can’t nail it down so neatly, but we can make a couple of observations. First, if publicity is a commonplace occurrence, and millionth-order ascriptions of belief to others is not a commonplace occurrence (as opponents of the Stalnaker-Lewis picture, including the author, are likely to insist), then it is not plausible that whenever something is public it is commonly believed. The salient rival hypothesis that to be public is to be true and commonly believed* is not so easy to dismiss. However, we have little positive reason to embrace this, as against intermediate-strength hypotheses such as: to be public is to be commonly believed*, and mutually believed to be mutually believed. I don’t see much prospect of pinning down a necessary and sufficient condition for publicity among the infinity of intermediate-strength principles here.

## Factivity: Its Source and a Route to Non-Factive Publicity

The result that publicity is a factive operator has troubled some readers of this paper.^24^ On the present account, something that is public between a small group of friends, for example, that Boris Johnson is the current UK Prime Minister, could change to not being public among them, without any of the friends’ attitudes changing. Given factivity, this could happen in virtue of sudden coup, resignation or death hundreds of miles away. Isn’t this the wrong result?

I think it is not a wrong result. There are both factive and non-factive senses in which a proposition can be public among a group (just as there are, for example, both factive and non-factive senses of related notions like “information” or “public information”). What our argument has done is to pin down that it is the *factive* sense, beliefs about which are suited to express the “treating as public among G” attitude that plays an important functional role from an insider’s, deliberative, perspective.

But if the insider’s perspective motivates and fixes the contours of a factive notion of publicity, what of the related, non-factive notion? The task of the current section is to show how an account of non-factive publicity, “publicity2”, can be developed in parallel to the argument given above, if we start from the outsider’s perspective rather than the insider’s.

Recall that two distinct elements are fused in the attitude I am calling “treating *p* as public”. It has a world-directed component: holding *p* fixed when we suppose the world to be this way or that, which amounts to believing *p*. It is this component that makes it a mistake to treat *p* as public when *p* doesn’t obtain. It also had a purely person-directed component: holding *p* fixed when one supposes oneself to be in some G-member’s shoes. I give this component its own label: “projecting *p* onto G”.

Consider this from the perspective of an insider. If a group member (who is aware that they are a group member) projects *p* onto G, they are committed, as a special case, to believe that they themselves believe *p*. They will be committed to a Moore-paradoxical combination of attitudes unless they do indeed believe that p. Splitting *treating-as-public* into belief and projection components would be psychologically artificial from the insider’s perspective. But when we consider the same situation from an outsider’s perspective, this changes. A non-member of G can perfectly reasonably project *p* onto G, while disbelieving *p* themselves. An outsider, informed that a coup has occurred, will project *Boris Johnson is PM* onto the group of friends who are ignorant of the coup, but will believe themselves that *Boris Johnson is not PM*.

The earlier implicit definition of publicity (or publicity1) said, in the current terminology: what it is to believe that *p* is public is to believe *p* and project *p* onto G. This expressed the insider’s take on publicity. I offer the following parallel implicit definition of “publicity2” that strips away the belief component: to believe that *p* is public2 is to project *p* onto G. Publicity2 is the outsider’s take on publicity.

One can now go to work on an account of publicity2 that parallels the earlier account of publicity. To adapt the earlier proofs, the key question will be: under what conditions is *projecting p onto G* correct? I say: it is necessary but not sufficient for this that all members of G also project *p* onto G; if all G members disbelieve *p*, but each projects *p* onto the others, then each will be mistaken, inaccurately representing others as believing *p*. What is necessary and sufficient for the correctness of projecting *p* onto G is this: all members of G project *p* onto G and also believe *p* themselves, i.e. all members of G treat *p* as public. It turns out that common belief in *p* entails it is public2 that *p*, which in turn entails common belief* in *p.* (The proof is given in the formal Online Appendix. The premises and derivations exactly parallel those in Sects. 2, 4 and 5.) Publicity2 is the nonfactive analogue to the concept of publicity (publicity1) that appeared in earlier sections.

Both the insider and outsider’s perspective are legitimate, and the naturalness of taking one or the other as one’s starting point varies according to theoretical focus. Moreover the notions of publicity associated with each allow easy approximations of the other. If one starts from the non-factive notion *publicity2*, one can recapture the factive notion of it being public/public information that *p* thus: *p* and it is public2 that *p*. If one starts from the factive notion of publicity/public information, one can approximate publicity2 thus: everyone in *G* believes that *p* is public*.* There is no need to force a choice between the two: we can and should acknowledge the good standing of both, underpinned by the parallel foundations developed in this paper.

## Group vs. Plural de re Common Belief

Before wrapping up, I want to highlight and evaluate one of the features of both characterizations of publicity I have argued for. This feature is a way in which my characterizations of publicity differ from the standard formal treatment that I sketched in Sect. 3. The feature is also the basis for an objection to the argument I gave in Sects. 2 and 4, an objection I here articulate and rebut.^25^

(I have carefully distinguished common belief and common belief* in all the discussion so far. But it will be annoying to have to keep track of all the superscripts below, and so for convenience in what follows I’ll make the assumption that common belief* is just common belief, by adding the Lewis-Stalnaker logical omniscience assumption that collapses the two notions. Nothing turns on this, and the reader can add the stars back in by hand as they wish.)

The feature in question is that common belief, as I have been working with it, is *group* common belief, whereas the standard formal treatments concern *plural de re* common belief. Group common belief requires each member of a group to believe quantified contents such as: *everyone in G believes that p, everyone in G believes that everyone in G believes that p,* etc. Plural de re common belief among Alice and Bob requires that: *Alice believes that p, Bob believes that p, Alice believes that Alice believes that p and Bob believes that p. Bob believes that Alice believes that p and Bob believes that p,* etc. So the contents of belief differ: one involves subjects quantifying over the members of some group; the other involves them ascribing beliefs de re to individuals. Group and plural de re common belief can easily come apart, even when the members of group G are exactly Alice and Bob. Suppose G is “the people in the room”. Alice may believe that all the people in the room heard the explosion, but not believe that she and Bob heard the explosion, because she does not believe that the other person in the room is Bob.

Just as it is plural de re common belief that is modelled by the standard formalism, it is plural de re common belief that is widely applied, for example, by game theorists. And one can see why: one thing that’s important to us in a non-isolated decision situation is who the other actors are—minimally, how many of them there are. Group common belief among G, for purely descriptive G (e.g. “the people in the room”) leaves room for a lot of uncertainty about these matters, and so doesn’t at all guarantee that we know the basic format of the games we are playing. However, one should not despair. Plural de re common belief is, after all, equivalent to a special case of group common belief: a case where G is “listiform”, for example: e.g. “the people who are identical to one of Alice or Bob”. So if my argument establishes a connection between publicity and group common belief, then it thereby establishes a connection between publicity and plural de re common belief, as a special case. Or so one might have thought.

Now to the problem for the argument. Premises (1) and (2) of the original argument each assert that a certain principle is a priori. This was based on the underlying claim that theses such as the following were a priori:

(INSTANTIATION EQUIVALENCE)

For all *x, x* treats *p* as public among G iff *x* believes that it is public in G that *p*.

This is a schematic principle—it says different things depending on what predicate we substitute for G. The objection will be that on many substitutions it is false that the resulting proposition is a priori. If the objection is right, then many instances of the argument above are unsound, and publicity will not *generally* entail common belief. To see the prima facie problem, replace “G” by the listiform term “those identical to one of Alice or Bob” or “those items in the set {Alice, Bob}”. These complex terms are built out of names for individuals. On orthodox assumptions, a sentence containing a name (outside of any quotational or intensional context) can only be true if the name is nonempty—in this case, if Alice and Bob exist. But (says the objector) it’s not an a priori matter whether Alice or Bob exists. So it seems that it can’t be a priori that the sentence or what the sentence says is true.

To be clear about the objection here: INSTANTIATION EQUIVALENCE may be true. But the charge is that only for purely descriptive substitutions for G will it be true and a priori. But aprioricity is required if it is to support premises (1) and (2). So in general, the argument in Sect. 4 will be unsound, in many instances. The objector has no problem with instances of the argument involving “purely descriptive” replacements for G—those not containing names or other a posteriori-existence-entailing expressions. But the kind of listiform replacements for G that are crucial to getting us to plural de re common belief make the argument unsound.

I say: even if this objection is correct so far as it goes, the residual (purely descriptive) sound instances of the argument are sufficient to give us everything we might want out of it. I also say: the objection is not correct. I am more confident of the first point than the second point, so I will first explain the work-around I favour, before briefly sketching the ways of responding to the objection directly.

Accordingly, I suppose first that the objection succeeds as stated. Instances of the argument involving listiform-style replacements for G are unsound, but many instances involving “purely descriptive” terms are good. Our task is to see how far the good instances take us.

Nothing in the discussion so far prevents it being public among G that those who satisfy G are exactly Alice or Bob. So long as G itself is purely descriptive (“the people, if any, in the room”), a good instance of the original argument will establish that it is common belief among the Gs that they are exactly Alice and Bob. Any listiform information about the character of the group that is truly public can therefore be accounted for within the restrictions that the objector imposes upon us. Now, this does not immediately allow us to recover plural de re common belief, strictly speaking.^26^ However, group common belief, plus group common belief of a plural de re identification of the members of the group, gives us everything we need to underpin theoretical applications of the notion. I suggested above, for example, that it might be thought important to attribute plural de re common belief to represent cases where agents take for granted the basic features of the game they’re playing—who the other agents are, how many of them there are, etc. When the de re identification of group members is itself part of what is commonly believed, then group members will indeed all be taking for granted these fundamental features of the framing of their decision problem. All the objection does is force us to move such information from the *form* of common belief to the *content* of common belief. This seems to me an independently attractive move.

I’ve made the case that in favourable circumstances, a restriction of the argument proper to purely descriptive identifications of the group still allows us to extract what we need. A second way of making trouble is to question whether circumstances are often-enough favourable. For example, many of our small group interactions do, intuitively, have a plural de re character. The natural characterization of Alice and Bob, as friends engaged in lively conversation, is that various things are public *between Alice and Bob.* That is, the predicate used to characterize the starting point scenario has exactly the listiform character that blocks the argument from being applied. The argument would obviously be far less interesting if it failed to apply to, and so said nothing about, these paradigmatic non-isolated decision situations.

In response, I submit that for Alice to treat-as-public *p among Alice and Bob* is ipso facto for her to treat-as-public *p* among G, where G is a suitable purely descriptive term. G may, for example, be: *the people identical either to whoever is Alice, or to whoever is Bob*, where “is Alice” (/ “Alicizes”) and “is Bob” (/ “Bobifies”) are understood as non-existentially-committing predicates that are satisfied only by Alice/Bob respectively. In such situations Alice will, I submit, also be treating as public among the Alicizers and Bobifiers the information that the Alicizers and Bobifiers are, exactly, Alice and Bob. Such descriptive purification of the listiform group is a trick that can always be applied. So in run-of-the-mill small group situations we will always be able to find a purely descriptive instance of the term on which the argument can be soundly run, which entails group common belief in *p*, and will also make plausible (given the setup) that there is group common belief in the relevant de re identification of the group members. The worry that the argument would not apply to these paradigmatic situations of common belief fades away.

The moves and countermoves above are conducted under the assumption that the original objection to instances of the argument involving de re content in the term “G” was a good one. My conclusion is that if the objection is good, we can work around it. But we may not need to be so concessive. The objection may itself rest on a mistake.

First, the objection as stated will not work if sentences containing empty names can be true. Many already think that it is only an artifact of formal languages that they disallow this.^27^ Traditional candidates for true sentences of featuring empty names include: “Pegasus does not exist” and “{*x*: *x* = Pegasus} is the empty set”. There are well known free logic formalisms that allow us to accommodate and regiment these natural seeming claims. If we handle empty names via a negative free logic, then the premises required to run the argument on a listiform group specification will be a priori, even if it is not a priori that Alice and Bob exist. So the argument will not be restricted to purely descriptive group specifications.

I find this line of response pretty plausible, so far as it goes. But I think this only defeats the letter of the objection, not its spirit. Plural de re common belief won’t follow from listiform group common belief alone, in a negative free logic setting. Classically, “everyone who is identical to N is F” entails “N is F”, but in the negative free logic setting that inference is enthymetic, and requires the additional premise “N exists”. The upshot is that in order to get from listiform group common belief to plural de re common belief, we will need to add as a premise that it is true and commonly believed that the various individuals on the list exist. Ultimately: the sort of information we needed to add to extend the scope of the argument under purely descriptive restrictions will also be needed here. It is not an independent response to the challenge. The main advantage of shifting to a free logic setting is that we remove the need to go searching for artificial purely descriptive correlates of listiform group specifications, since in the relevant sense, the lists themselves will be “purely descriptive”.

A second response cuts deeper, but is far more philosophically controversial. The objection we have been discussing that it is not a priori that Alice and Bob (for example) exist. In recent years, philosophers have argued (from the armchair) that there is no such thing as contingent existence.^28^ According to this line of thought, what we usually think of as possibilities where Caesar never existed, for example, are really possibilities where Caesar exists but has none of the properties usually attributed to him—not even concrete existence in space and time. I was worried by the original argument because it seemed that once you had mentioned Alice and Bob in a sentence, then on orthodox accounts the sentence (and what the sentence expresses) would turn out to be false at epistemically possible scenarios where Alice doesn’t exist. But if such scenarios are a priori impossible, this worry falls away. Now, even if Alice and Bob’s existence is a priori, it is perfectly epistemically possible that Alice and Bob are not thinkers, but rocks or abstracta lacking any distinctive properties at all (the latter being the necessitist’s proxy for cases of nonexistence). But unlike the case of nonexistence, this does not undercut the premises of the argument I gave. The premises assumed to be a priori are material conditionals. They say (for example) that if something is public among Alice and Bob, then Alice and Bob hold such-and-such attitudes. Such conditionals can be a priori true in a scenario in which Alice and Bob are rocks or abstracta, so long as the relevant rocks or abstracta lacking attitudes towards *p* is matched by *p* failing to be public among them, in the relevant scenario.

The arguments against contingent existence are usually run for metaphysical modalities, not epistemic ones, and so work would have to be done to extend these arguments if this is to be the response. I am not going to do that work here. I’m only 50/50 on whether the considerations extend to the epistemic modalities, and less than 50/50 on whether we should accept the original necessitarian thesis. So I’m not very confident that this is the right line of resistance. But it is a direction worth exploring.

Whatever way we go, there is no serious objection to the soundness of the instances of the argument that matter for its application from the considerations floated at the beginning of this section.

## Publicity as a Simple Concept

The view that emerges is as follows. We have a concept, publicity (i.e. publicity1, above), which we use to identify a class of propositions that have a special role in thinking about the attitudes of a group. Beliefs about what is public are intimately linked to (identified with) what we treat as public. If something is public this requires, a priori, that it is believed by all members of the group, that all members of the group are committed to believe it is believed by all members, and so on. In short, a proposition being public requires that it is commonly believed*.

There is nothing particularly cognitively complex going on when we judge: such-and-such is public. In particular, there is no implication that we are thinking through some vast infinite conjunction. Common belief* is defined that way, but (/true) common belief* and publicity are distinct concepts, one complex, one simple. That is so whether or not they are a priori equivalent. Nor is there anything particularly complex in ascribing this belief to others. To think that everyone in the room believes that it’s *public among those in the room* that the meeting is running over is no more demanding, cognitively or epistemically, than to think that everyone in the room believes that it is *annoying to everyone in the room* that the meeting is running over.

Suppose we are in one of those situations where we all think that everyone believes that it’s public among us that the meeting is running over. So long as the meeting *is* in fact running over, then on the assumptions in the last section it is true that the meeting is running over is public among us. This is because just as (PUBLIC) combines with other assumptions to give (INTROSPECTION), the new principle (STRONG PUBLIC) biconditional argued for in Sect. 5 combines with other assumptions to give:

(STRONG INTROSPECTION)

It is public in G that *p* if and only if *p* and everyone in G believes that it is public in G that *p*.^29^

Even if we reject the key assumptions of the last section, we should not think that it is hard to get true beliefs about what is public on the grounds that it entails common belief*.

This might seem strange. Publicity is a very strong property. Since it entails common belief*, it requires that each group member commit to rule out each of the following empirical possibilities: that *p* be mutually believed without being mutually mutually believed, that *p* be mutually mutually believed without being mutually mutually mutually believed, etc. By believing something to be public, we rule out at a stroke all these ways in which publicity can fail. That could lead to puzzlement about how such a belief could ever be responsibly formed, without performing some heroic supertask of checking the higher-order mental states of others. But the point is that we expect publicity to be made true not by others first taking a stance on the target proposition *p*, and also considering and taking a further stand on the proposition that everyone believes *p*, and so forth. We should expect it to be true in virtue of others taking a stand on the single question of whether it is public that *p*.

## Conclusion

In various parts of the literature in social ontology and collective intentionality, a lot of emphasis is put on a concept of information being “public”. Common belief (or some close variant), defined via the kind of infinite conjunction of iterated attitude-ascriptions is the default way to model the notion—perhaps even the basis for an analysis of it. But rarely was there much discussion about why we needed to handle publicity in this fashion. Looking at the various discussions in Gilbert (1989) on joint commitment, in Bratman (2014) within the analysis of shared intention, in Lewis (1969) as a part of the analysis of convention, or in Stalnaker (2014) in the analysis of the common ground of a conversation, one struggles to find direct arguments to this end.

What does emerge from the literature is a conception of publicity/common belief as a notion peculiarly relevant to deliberation (perhaps collective deliberation) in cases of non-isolated action. This has been my starting point in this paper. I’ve constructed an argument that a notion of publicity must entail one of those close relatives of common belief, if it is to play the practical role assigned to it. Indeed, I think we have plausible grounds for taking it to be a notion strictly between the property of (true) common belief and the notion I call (true) common belief*.

The considerations given here deliver what I call group common belief, not plural de re common belief. As has become evident in the last section, the relation between these two notions is not straightforward. I have argued, however, that group common belief gives us something that is as near to plural de re common belief as we require for the notion to be theoretically interesting.

My goal in this paper has been to present the best case for a link between common belief and publicity (better: between common belief and two disambiguations of publicity, indexed to the insider’s and outsider’s perspective). I have not engaged directly with extant arguments which would say that common belief(*) cannot be related to publicity in the way I have derived, if any proposition is to count as public between creatures like us. Lederman (2018a) has a sceptical argument to this effect, for example, and while I have things to say about it, this is not the place to say them. If one is convinced that the conclusion of the argument in the paper is incorrect, it still has value. One will then regard the overall argument as a reductio of the premises I have identified. One might think, for example, that the mistake was to think that there is a coherent notion of treating-as-public at all. One might alternatively trace the error to the assumption that the attitude of treating-as-public is identified with a belief with a specific (publicity-involving) content. Whether this is the right way to react to the argument put forward in this paper, or whether one should accept the premises and embrace the conclusion, is a matter for another occasion.

## Supplementary Information

Below is the link to the electronic supplementary material.

Supplementary file1 (PDF 4457 kb)

## Footnotes

1 In this I align, for example, with Ganson (2008), Ross and Schroeder (2014). Bratman (1992) holds that an attitude he calls *acceptance* plays this role. Stanley (2005) is a representative of a cluster of views that hold that *knowledge* plays this role. My starting assumption means that the rest of this paper is conducted in terms of common belief and related notions. If one started with a view on which *acceptance* plays a role in isolated cases, then it would be natural to focus instead on common acceptance (as in Stalnaker 2014) or common knowledge (as in Lederman 2018a, b).

2 I’ll be using the variable G to range over groups throughout the main text (for present purposes, groups are understood liberally, as any set of persons). The main text instances of G will be syntactically singular, associated with singular terms like ‘the people in the room’. In the formalism in footnotes, and in the formal appendix, I use G as a predicate, associated with predicates such as ‘is a person in the room’. From a predicate F of people, we can form the singular term for a group, ‘the Fs’. From a singular term for a group of people, n, we can form the predicate of people, ‘is in/is a member of n’. The choices are intended to be purely presentational decisions, with nothing of substance hanging on them.

3 One source for this kind of iterated notion is Lewis (1969). It was independently rediscovered by others, including Aumann, from where it led to the development of a rich formal literature. See Fagin et al (1995). Cubitt and Sugden (2003) formalize Lewis’s notion and contrast it with the now-standard approach.

4 The general idea of characterizing a notion related to common belief by relaxing the clauses to require something less than full belief is already present in Lewis (1969). See also Gilbert (1989)’s appeals to “smooth-reasoning counterparts” to our actual selves in her favoured definition.

5 I owe the terminology to Lederman (2018b).

6 In symbols:$\forall x\left({T}_{x}^{G}p\supset {B}_{x}p\right)$. I’ll keep the exposition in the main text informal, but give these regimentations in footnotes. A formalization of the derivations from these premises is given in the Online appendix.

7 In symbols: $\forall x\left({\text{correct}}\left[{B}_{x}p\right]\equiv p\right)$. The correctness operator is not factive, or else this biconditional would fail in worlds where *p* obtains but *x* does not believe it to obtain. The idea is that we can still evaluate a belief-that-*p* as correct or not at worlds where nothing is believed—this corresponds to the truth-conditions of the belief being satisfied at the world in question. A referee notes an analogy between BELIEF CORRECTNESS and the Tarski biconditionals (if one replaced the operator “*x* believes that” with a quotation-forming operator, they would be identical), and worries about liar-like pathologies. The fact that quotation is a term-forming operator is crucial to the standard ways of forming liar sentences (L: = “L is not true”) and is not obvious how to replicate here. It’s worth noting that I will only be using instances of BELIEF CORRECTNESS so if necessary, I would restrict this principle, and the conclusion derived, to non-pathological instances of *p*.

8 In symbols: $\forall x(({\text{correct}}[{T}_{x}^{G}p]\supset \forall y(Gy\supset {T}_{y}^{G}p))$. An important possible strengthening is where we strengthen the embedded conditional to a biconditional. I end up rejecting this strengthening in a section below, and suggesting a different biconditional formulation.

9 In symbols: $$\forall x\left({\text{correct}}\left[{T}_{x}^{G}p\right]\equiv {\text{correct}}\left[{B}_{x}{P}^{G}p\right]\right)$$.

10 In symbols: $\forall x\left({T}_{x}^{G}p\equiv {B}_{x}{P}^{G}p\right)$. Suppose that the logic of the correctness operator is such that it is a priori closed under a priori equivalence (or, stronger, under consequence). Then, as a referee for this journal pointed out, INSTANTIATION EQUIVALENCE would follow from CORRECTNESS EQUIVALENCE. That closure principle is probably too strong. It would mean that in order for *x* to believe that *p* is public among G, the following must be the case: ${\text{correct}}\left[\left(x\right){B}_{x}{P}^{G}p\right]$. It’s hard to know what the latter statement says. A plausible refined closure principle would restrict it to cases of a priori consequence between atomic attitude reports, or perhaps atomics and their negations. Note that since *x* treating-as-public *p* a priori entails *x* believing *p* (by BELIEF ENTAILMENT), closure of correctness over attitude-literals (or closure of incorrectness over attitude-atomics) would tell us that a condition of the correctness of *x* treating-as-public *p* is the correctness of *x* believing that *p*. By BELIEF CORRECTNESS we get that a condition of the correctness of x treating-as-public p is that p obtain. That principle will be picked up in a later section.

11 In symbols: ${P}^{G}p\supset \forall x\left(Gx\supset {T}_{x}^{G}p\right)$. If we had a biconditional version of TREATS CORRECTNESS, then the biconditional form of PUBLIC would follow. Lederman takes the biconditional version of PUBLIC as a definition of the operator “it is public that”. If we had this, and BELIEF CORRECTNESS and TREATS CORRECTNESS, we can get one direction of CORRECTNESS EQUIVALENCE. The other direction would follow if we strengthened TREATS CORRECTNESS to a biconditional.

12 In symbols: $${P}^{G}p\supset \forall x\left(Gx\supset {B}_{x}{P}^{G}p\right)$$

13 In symbols: ${P}^{G}p\supset \forall x\left(Gx\supset {B}_{x}p\right)$. The “a priori reasoning from a priori concepts establishes a prioricity” corresponds to the assumption CLOS in the formal appendix.

14 Formulating common belief as an infinite conjunction requires we use an infinitary language. That reflects faithfully the informal characterization of common belief. But the technical treatment of infinitary languages can be subtle. In what follows, for “*q* entails that *p* is commonly believed” one can usually read “*q* entails *r,* for each conjunct *r* of the usual infinitary definition of common belief”. In effect, I substitute quantification in the metalanguage for an infinitary object language. The formal appendix uses an infinitary rule, rather than define properties via infinite conjunctions.

15 E.g. Stalnaker 1984, Lewis, 1986. This is also a common modelling assumption in the tradition reported by Fagin et al (1995), but importantly, Stalnaker and Lewis endorse it as a true claim about ordinary mortals, not an idealizing assumption.

16 This corresponds to the principle COMM in the formal appendix.

17 The formal appendix deploys an infinitary introduction-rule for common belief*, which if we regard common belief* as the infinite conjunction above could be derived from infinitary conjunction-introduction. The advantage of using the former rule is that it avoids having to formalize the reasoning using infinitely-long sentences.

18 The formal appendix reconstructs this reasoning in full detail. As well as A PRIORI CLOSURE OF COMMITMENT (COMM) and the principle CLOS mentioned previously. It also uses a further principle not made explicit in this informal presentation: that what is a priori is a priori apriori (ITER).

19 Again, in this I follow in the footsteps of Lewis (1969), Gilbert (1989) and others. The exact relationship between my notion of common commitment to believe and these precedents I won’t analyze here, but I will emphasize that on my telling, one can be committed to believing things one has no (normative) reason to believe. That seems to be a difference with Lewis’s approach at least, though it’s not clear to me whether his followers (e.g. Cubitt and Sugden (2003)) follow him in this respect.

20 In symbols: $$\left({C}^{G}p\supset \forall y\left(Gy\supset {T}_{y}^{G}p\right)\right)$$.

21 This is the same line of thought that led us to the earlier BELIEF ENTAILMENT principle. To repeat a point made in the earlier footnote 10: if we accepted the closure of incorrectness under a priori consequence over atomic attitude reports, then BELIEF ENTAILMENT plus BELIEF CORRECTNESS would entail “factual treats correctness”, i.e. *x* treating *p* as public among G is correct only if *p*.

22 In $$\mathrm{symbols}: \forall x(correct[{T}_{x}^{G}p]\equiv p\&\forall y(Gy\supset {T}_{y}^{G}p).$$

23 In symbols: ${P}^{G}p\equiv p\wedge \forall x\left(Gx\supset {T}_{x}^{G}p\right)$. Note that STRONG PUBLIC is equivalent to STRONG TREATS CORRECTNESS relative to premises set out in the earlier section.

24 In particular, a referee for this journal worried that it leads us to counterintuitive consequences. I am grateful for the instructive exchange that led to the current section—though I know that they remain somewhat unconvinced!

25 I thank Harvey Lederman for pressing me on this issue.

26 The interested reader might want to explore what assumptions we need to add to the setting to derive plural de re common belief in *p* from group common belief among G that *p* plus group common belief among G that G contains all and only the individuals in a certain list. As far as I can see some version of multi-premise closure added to “commitment to believe” would be necessary in order to get this. To see this, note that group common belief among that G = {Alice, Bob} would entail both that Alice and Bob both believe that G = {Alice, Bob}, and both believe that everyone in G believes that G = {Alice, Bob}. If they believed the utterly obvious consequences of these two beliefs, then they would believe that Alice and Bob believe that G = {Alice, Bob}. If all the above reasoning is a priori, then this can feed into the kind of recursive derivation given above.

27 See Nolt (2018) for a survey of this issue.

28 A classic starting point for this is Williamson (2002). See also Williamson (2013).

29 In symbols: ${P}^{G}p\equiv p\wedge \forall x\left(Gx\supset {B}_{x}{P}^{G}p\right)$. The derivation of this is given in the online appendix, in the section formalizing Sect. 5.
