# Supplementary material for: Publicity and Common Commitment to Believe
Source: Erkenntnis. 2021 May 24;88(3):1059–80. doi: 10.1007/s10670-021-00393-x (PMC10024650; doi:10.1007/s10670-021-00393-x)
Supplement: Supplementary file 1 — Supplementary file1 (PDF 4457 kb) [file 10670_2021_393_MOESM1_ESM.pdf]

APPENDIX

This appendix presents a minimal formalization of the derivations that appear in the main text. It will not attempt to identify a single favoured "formal system" (formal language, proof theory, model theory) for the target notions—an interesting project, but one for another day.[1] Rather, it will set out the basic vocabulary required and exhibit the inferential steps involved. This will put readers in a position to inspect formalizations of the derivations and directly evaluate their cogency, or bring to bear their favourite formal system and check that it validates the reasoning.

I assume we are working within a language extending the language of first-order predicate logic. $\supset$ and $\equiv$ will stand for the material conditional and biconditional respectively. The language includes in addition a number of higher order relation symbols. Note however that only first order quantification will be used within the derivation to follow, and quantification into the scope of the higher order relation symbols is used only for one symbol: $\mathsf{correct}$.

The initial list of higher order relational symbols are as follows, with the syntactic types of their argument places indicated by the schematic use of an objectual variable x, one-place predicate letter G, and sentence letter q.

- $B_x q$: x believes that q.
- $B^*_x q$: x is committed to believe that q.
- T^G_x: x treats G as public among the group G.
- $P^G q$: it is public among G that q
- $\textsc{ap}q$: it is a priori that q
- $\mathsf{correct}[q]$: the attitude reported by q is correct.

Additional higher order relation symbols are introduced below where relevant.

The inferential steps used in the formalized derivations below are of two kinds. First, there are inference rules involving the higher-order symbols. Second, there are substitution instances of familiar elementary first-order validities such as modus ponens, conjunction elimination/introduction, weakening the consequent of a material conditional, etc.
The inferential rules of the first kind that we will use include:

ITER: $\textsc{ap}p \models \textsc{ap}[\textsc{ap}p]$

COMM: $\textsc{ap}[p \supset q] \models B_x p \supset B^*_x q$

CLOS: $\Gamma \models q \Longrightarrow \textsc{ap}(\Gamma) \models \textsc{ap}q$

In the above, $\textsc{ap}(\Gamma)$ is the set resulting from prefixing $\textsc{ap}$ to each element of the set Γ. Some further rules will be added to this list in discussing the derivations of section 5.

It would try the reader's patience to explicitly label every inferential move of the second kind that I rely upon. I will instead use the generic tag 'logic' to indicate that elementary moves of this kind are being used. There are however several moves I use repeatedly, and cite in the derivations to follow:

Transitivity of equivalence $\forall x(\phi \equiv \psi), \forall x(\psi \equiv \chi) \models \forall x(\phi \equiv \chi)$

Transitivity of implication $\alpha \supset \forall x(\phi \supset \psi), \forall x(\psi \supset \chi) \models \alpha \supset \forall x(\phi \supset \chi)$

Substitution of equivalents Various forms used including:

$\forall x(\phi \equiv \psi), \forall x(\phi \supset \chi) \models \forall x(\psi \supset \chi)$
$\forall x(\phi \equiv \psi), \forall x(\chi \supset \phi) \models \forall x(\chi \supset \psi)$
$\forall x(\phi \equiv \psi), \forall x(\chi \supset \forall y(\rho \supset \phi)) \models \forall x(\chi \supset \forall y(\rho \supset \psi))$
$\forall x(\phi \equiv \psi), \forall x(\chi \equiv \forall y(\rho \supset \phi)) \models \forall x(\chi \equiv \forall y(\rho \supset \psi))$

[1]See Fagin et al (1995) for an introduction to the standard multimodal logic treatment of common belief and related notions.

I'll use these terms also for obvious logical variants of them (e.g. those omitting vacuous quantifiers). The general pattern behind the final family is to allow substitution of equivalents in any 'first order' context, i.e. contexts outside the scope of the higher order relation symbols. Substitution of material equivalents within the scope of higher order operators runs the risk of invalidity when those symbols generate (hyper)intensional contexts, but there is no such danger in the instances listed.

Formalization of the derivation in section 2

The five main premises of section 2 are:

BE: BELIEF ENTAILMENT:
$\forall x(T^G_x p \supset B_x p)$

BC: BELIEF CORRECTNESS:
$\forall x(\mathsf{correct}[B_x p] \equiv p))$

TC: TREATS CORRECTNESS:
$\forall x(\mathsf{correct}[T^G_x p] \supset \forall y(Gy \supset T^G_y p))$

CE: CORRECTNESS EQUIVALENCE:
$\forall x(\mathsf{correct}[T^G_x p] \equiv \mathsf{correct}[B_x P^G p])$

IE: INSTANTIATION EQUIVALENCE:
$\forall x(T^G_x p \equiv B_x P^G p)$

I call the result of prefixing AP to one of the above its 'strengthening', and e.g. use AP(BE) to denote the strengthened form of BE. Despite the name, note we have not yet made the assumption that AP is factive, though that assumption will be introduced later.

The derivation in section 2 is formalized as follows, (2.3) is the principle labelled in the main text PUBLIC, (2.4) is INTROSPECTION and (2.5) is PUBLIC BELIEF ENTAILMENT.

2.1 $\forall x(\mathsf{correct}[T^G_x p] \equiv P^G p)$ [from CE and BC, transitivity of equivalence],

2.2 $\forall x(P^G p \supset \forall y(Gy \supset T^G_y p))$ [from 2.1 and TC, substituting equivalents],

2.3 $P^G p \supset \forall y(Gy \supset T^G_y p))$ [from 2.2, logic],

2.4 $P^G p \supset \forall y(Gy \supset B_y P^G p))$ [from 2.3 and IE, substituting equivalents],

2.5 $P^G p \supset \forall y(Gy \supset B_y p))$ [from 2.3 and BE, transitivity of implication].

The derivation just given shows, for $1 \leq k \leq 5$:

BE, BC, TC, CE, IE $\models$ 2.k

And so by CLOS we have:

AP(BE), AP(BC), AP(TC), AP(CE), AP(IE) $\models$ AP(2.k)

So from the strengthened main premises we get similarly strengthened conclusions. It is AP(2.4) and AP(2.5) that will be used below.

Formalization of the derivation in section 4

The sentences to be proved from AP(2.4,2.5) are:

4.1 $\text{AP}[P^G p \supset \forall x(Gx \supset B_x p)]$

4.2 $\text{AP}[P^G p \supset \forall x(Gx \supset B^*_x[\forall y(Gy \supset B_y p)])]$

4.3 $\text{AP}[P^G p \supset \forall x(Gx \supset B^*_x[\forall x(Gy \supset B^*_y[\forall z(Gz \supset B_z p)])])]$

And more generally for all k:

4.k $\text{AP}[P^G p \supset \forall x_1(Gx_1 \supset B^*_{x_1}[\forall x_2(Gx_2 \supset B^*_{x_2}[\ldots[\forall x_k(Gx_k \supset B_{x_k}p)]\ldots])])]$

We start the proof with a lemma. COMM tells us that $\text{AP}[p \supset q] \models B_x p \supset B^*_x q$. Whence by CLOS we get: $\text{AP}[\text{AP}[p \supset q]] \models \text{AP}[B_x p \supset B^*_x q]$. Using ITER, we derive the following:

(*): $\text{AP}[p \supset q] \models \text{AP}[B_x p \supset B^*_x q]$

The derivation of (4.1),(4.2) and (4.3) can then proceed as follows:

4.1.1 $\text{AP}[P^G p \supset \forall y(Gy \supset B_y P^G p))]$ [AP(2.4) restated]

4.1 $\text{AP}[P^G p \supset \forall x(Gx \supset B_x p)]$ [AP(2.5) restated]

4.2.1 $\text{AP}[\forall y(B_y P^G p \supset B^*_y[\forall x(Gx \supset B_x p)]]$ [from 4.1 by (*)]

4.2 $\text{AP}[P^G p \supset \forall y(Gy \supset B^*_y[\forall x(Gy \supset B_x p)])]$

[from 4.1.1, 4.2.1, transitivity of implication, CLOS]

4.3.1 $\text{AP}[\forall z(B_z P^G p \supset B^*_z[\forall y(Gy \supset B^*_y[\forall x(Gy \supset B_x p)])]]$ [from 4.2 by (*)]

4.3 $\text{AP}[P^G p \supset \forall x(Gx \supset B^*_x[\forall x(Gy \supset B^*_y[\forall z(Gz \supset B_z p)])])]$

[from 4.1.1,4.3.1, transitivity of implication, CLOS]

The pattern exhibited in the final two pairs of steps can be extended indefinitely. The inductive step to establish (4.k) for all k is in a metalinguistic induction is the following. Assuming we have already derived the k-1th instance:

4.(k-1) $\text{AP}[P^G p \supset \forall x_2(Gx_2 \supset B^*_{x_2}[\forall x_3(Gx_3 \supset B^*_{x_3}[\ldots[\forall x_k(Gx_k \supset B_{x_k}p)]\ldots])])]$

Applying inference rule (*) we obtain:

4.k.1 $\text{AP}[\forall x_1(B_{x_1} P^G p \supset B^*_{x_1}[\forall x_2(Gx_2 \supset B^*_{x_2}[\forall x_3(Gx_3 \supset B^*_{x_3}[\ldots[\forall x_k(Gx_k \supset B_{x_k}p)]\ldots])])])]$

From 4.1.1 and 4.k.1 we appeal to transitivity of implication and CLOS to finish the inductive step:

4.k $\text{AP}[P^G p \supset \forall x_1(Gx_1 \supset B^*_{x_1}[\forall x_2(Gx_2 \supset B^*_{x_2}[\ldots[\forall x_k(Gx_k \supset B_{x_k}p)]\ldots])])]$

The infinitary rules for C*

We now add the higher order relation symbols

- $C^G p$: it is commonly believed in G that p
- $C^{*G} p$: there is a common commitment to believe that p among G.

Let $M^G p := \forall x(Gx \supset B_x p)$, and set M^G_k to be the result of k iterations of M^G. In parallel fashion, we set $M^{*G} p := \forall x(Gx \supset B^*_x p)$, and let M^{*G}_k be the result of k iterations of M^{*G}. We now assume the following infinitary inference rule:[2]

C*-intro $\{M^{*G}_k M^G p\}_{0<k<\infty} \models C^{*G} p$

[2]This is one of a package of introduction and elimination rules we might assume:

C-intro $\{M^G_k p\}_{0<k<\infty} \models C^G p$

C-elim$_k$ $C^G p \models M^G_k p$

Label infinitary/infinite set of inference rules:

C*-intro $\{M^{*G}_k M^G p\}_{0<k<\infty} \models C^{*G} p$

C*-elim$_k$ $C^{*G} p \models M^{*G}_k M^G p$

In the main text, the gloss on C and C^* is as the infinite conjunctions $\bigwedge_{0<k<\infty} M^G_k p$ and $\bigwedge_{0<k<\infty} M^{*G}_k Mp$. The infinitary rules above would then be special instances of infinitary conjunction introduction and elimination. By positing the single infinitary rule needed, we sidestep having to extend our formal language to include infinite sentences.

Two further assumptions are used: an infinitary cut metarule, and a further very natural constraint on the logic of AP:

CUT: $\{\Gamma \models \psi_k\}_{0<k<\infty}, \{\psi_k\}_{0<k<\infty} \models \chi \Longrightarrow \Gamma \models \chi$

FACT: $\text{AP}p \models p$

In this notation, the conclusion of the last section, (4.k) can be rewritten succinctly as $\text{AP}[BE, BC, TC, CE, IE] \models \text{AP}[P^G p \supset M_k^{*G} M^G p]$, for each k.

By FACT, we get:

$$\text{AP}[BE, BC, TC, CE, IE] \models P^G p \supset M_k^{*G} M^G p$$

and so, for each k (by the validity of modus ponens):

$$\text{AP}[BE, BC, TC, CE, IE], P^G p \models M_k^{*G} M^G p$$

applying C*-intro, we get:

$$\text{AP}[BE, BC, TC, CE, IE], P^G p \models C^* p$$

and so by conditional proof:

$$\text{AP}[BE, BC, TC, CE, IE] \models P^G p \supset C^* p.$$

Using CLOS and ITER we derive:

$$\text{AP}[BE, BC, TC, CE, IE] \models \text{AP}[P^G p \supset C^* p.]$$

I note that the three assumptions introduced here are used only to support a succinct formulation of the results of the last section, and analogues thereof (5.7, 6.7), and so are not central to the main results.

Formalization of the derivation in section 5

Section 5 adds the following assumptions:

CB: COMMON BELIEF:
$C^G p \supset \forall x(Gx \supset (T_x^G p))$

STC: STRONG TREATS CORRECTNESS:
$\forall x(\mathsf{correct}[T_x^G p] \equiv (p \wedge \forall y(Gy \supset T_y^G p))$

The main derivation largely parallels that in section 2, replacing appeal to TC with appeal to STC. 5.3 is labelled STRONG PUBLIC in the main text. 5.4 is labelled STRONG INTROSPECTION in section 8 of the main text. 5.5 reports the factivity of the publicity operator.

5.1 $\forall x(\mathsf{correct}[T_x^G p] \equiv P^G p)$ [from CE and BC, transitivity of equivalence],

5.2 $\forall x(P^G p \equiv (p \wedge \forall y(Gy \supset T_y^G p))$ [from 5.1 and STC, transitivity of equivalence],

5.3 $P^G p \equiv (p \wedge \forall y(Gy \supset T_y^G p)))$ [from 5.2, logic].

5.4 $P^G p \equiv (p \wedge \forall y(Gy \supset B_y P^G p)))$ [from 5.3 and IE, subst of equivalents]

5.5 $P^G p \supset p$ [from 5.3, logic.]

By ITER and CLOS we can prefix AP to each 5.k, deriving the resulting formulae in each case from AP{BE, BC, STC, CE, IE, CB}. We further argue:

5.6 $(p \wedge C^G p) \supset P^G p$ [5.3, logic, transitivity of implication]

5.7 $P^G p \supset (p \wedge C^{*G} p)$ [see below]

5.7 follows from the results of the last section, which rest on AP{BE, BC, STC, CE, IE, CB}, together with 5.5 and logic. Again CLOS and ITER allow us to also derive all these conclusions with AP prefixed.

Formalization of the derivation in section 6

We add the higher order relation symbol, P^G_* glossed in the main text as 'publicity2', and $\textsc{proj}^G_x p$, glossed in the main text as 'x projects p onto G'. For the purposes of the derivation in this section, we do not need the earlier assumptions TC/STC, CE, IE or BE. Instead we use the following analogues (which can be used to rederive some of the original premises):

SPC: STRONG PROJ CORRECTNESS:
$\forall x(\mathsf{correct}[\textsc{proj}^G_x p] \equiv \forall y(Gy \supset \mathrm{T}^G_y p)))$

CEP: CORRECTNESS EQUIVALENCE for PROJ:
$\forall x(\mathsf{correct}[\textsc{proj}^G_x p] \equiv \mathsf{correct}[B_x P^G_* p]))$

IEP: INSTANTIATION EQUIVALENCE for PROJ:
$\forall x(\textsc{proj}^G_x p \equiv B_x P^G_* p))$

PTC: PROJ/TREATS CONNECTION:
$\forall x((\textsc{proj}^G_x p \wedge B_x p) \equiv T^G_x p)$

The derivation then proceeds as follows:

6.1 $\forall x(\mathsf{correct}[\textsc{proj}^G_x p] \equiv P^G_* p)$ [from CEP and BC, transitivity of equiv],

6.2 $P^G_* p \equiv \forall y(Gy \supset T^G_y p)))$ [from 6.1 and SPC, transitivity of equiv, logic],

6.3 $P^G_* p \equiv \forall y(Gy \supset B_y p \wedge \textsc{proj}^G_y p)))$ [from 6.2 and PTC, subst equivalents],

6.4 $P^G_* p \supset \forall y(Gy \supset B_y P^G_* p))$ [from 6.3 and IEP, subst equivalents, logic],

6.5 $P^G_* p \supset \forall y(Gy \supset B_y p))$ [from 6.3, logic].

This shows, for $1 \leq k \leq 5$:

BC, SPC, CEP, IEP, PTC $\models$ 6.k

And so by CLOS we have:

$\textsc{ap}$\{BC, SPC, CEP, IEP, PTC\} $\models$ $\textsc{ap}$(6.k)

Note that $\textsc{ap}$(6.4) and $\textsc{ap}$(6.5) have exactly the same form as $\textsc{ap}$(2.4) and $\textsc{ap}$(2.5). The former pair simply have the operator P^G_* where the latter has P^G. If we make the substitution of P^G_* for P^G throughout the formalized derivation in section 4, it remains sound, relative to the (strengthening with $\textsc{ap}$ of the) assumptions used in this section. Adding C*-intro, FACT and CUT, this reasoning establishes the lower bound 6.7, below. The corresponding upper bound, 6.6, follows directly from CB and 6.2.

6.6 $C^G p \supset P^G_* p$ [from CB and 6.2, transitivity of implication, logic]

6.7 $P^G_* p \supset C^{*G} p$ [from $\textsc{ap}$6.4,$\textsc{ap}$6.5, reasoning pattern of section 4, C*-intro, infinitary cut]

CLOS again ensures that from strengthened forms of each of the premises used in this derivation, we may prefix $\textsc{ap}$ to 6.6 and 6.7.
